# An Extremely Rare Case of Anomalous Left Main Coronary Artery Originating From Single Right Coronary Ostium Managed Using Heart Team Approach

**DOI:** 10.7759/cureus.8879

**Published:** 2020-06-28

**Authors:** Hina Akbar, Sobia Akbar, Sana Akbar, Rehan Kahloon

**Affiliations:** 1 Internal Medicine, University of Tennessee Health Science Center, Memphis, USA; 2 Internal Medicine, Regional One Hospital, Memphis, USA; 3 Internal Medicine, Postgraduate Medical Institute, Lahore, PAK; 4 Endocrinology, George Washington University, Washington, DC, USA; 5 Cardiology, Erlanger Health System/University of Tennessee College of Medicine, Chattanooga, USA

**Keywords:** coronary artery anomalies, coronary ostium, cardiac catheterization, chest pain, unstable angina, coronary artery disease, multi-vessel disease

## Abstract

Anomalies of coronary artery origin are rare, difficult to diagnose using conventional testing methods and extremely challenging to eventually manage once diagnosed. The risk of adverse outcomes increases as such patients age and develop atherosclerosis in such vessels. A comprehensive and multidisciplinary approach may be required to best manage such difficult cases.

We present a case of a 65-year-old female with symptoms of chest pain concerning for unstable angina. She also complained of occasional diaphoresis and dizziness. Physical examination revealed a regular heart rhythm with no vascular bruits. An electrocardiogram (EKG) only showed normal sinus rhythm and left axis deviation. Non-invasive testing included an echocardiogram, which showed multiple wall motion abnormalities. A diagnostic cardiac catheterization via right radial artery approach was performed to delineate her coronary anatomy and rule out ischemic etiology. This led to diagnosis of anomalous coronary anatomy with an anomalous left main coronary artery from single right coronary ostium. Furthermore, it showed significant obstructive multi-vessel coronary artery disease involving distal left main artery, proximal left anterior descending artery, left circumflex and right coronary arteries. The patient had a right dominant system with absent left coronary cusp. Percutaneous vs surgical revascularization options were considered. Given high Syntax score and acceptable Society of Thoracic Surgeons (STS) risk, Heart Team approach was pursued and the patient was referred for multi-vessel surgical revascularization.

## Introduction

Congenital coronary artery anomalies, including anomalous origin of a coronary artery, can manifest as life-threatening conditions, such as myocardial infarction or arrhythmia, and may even lead to sudden death. Diagnosing patients early on may be difficult because children and adolescents are often asymptomatic and first presentation may be sudden death or sudden cardiac arrest. Such anomalous origin arteries can also develop atherosclerotic lesions later on in life with patients having atherosclerotic cardiovascular disease (ASCVD) risk factors [1-3]. Acute plaque rupture and complete thrombosis in these arteries can result in acute coronary syndrome as well [4]. A combination of absent left coronary ostium and anomalous left main coronary artery with a single right coronary ostium is an extremely rare finding [5,6].

An aortic root angiogram during routine coronary angiography via femoral or radial artery access can help delineate the coronary anatomy showing absence of left coronary ostium. High-risk characteristics are most commonly assessed using two-dimensional (2D) echocardiogram (echo) or cardiac CT. 

Currently, there is significant variability in decision making regarding management of patients having anomalous coronary arteries with ASCVD. Future research is needed to help determine the best way to identify at-risk patients and which treatment is the safest and most efficacious once atherosclerotic disease sets in.

## Case presentation

A 65-year-old female with a history significant for tobacco use, chronic obstructive pulmonary disease (COPD), tobacco abuse, sarcoidosis, hypertension, hyperlipidemia and non-insulin-dependent diabetes mellitus presented for the first time to outpatient cardiology clinic for evaluation of her chest pain. She had initially seen her primary care physician in the outpatient setting and was ruled out to have pulmonary, musculoskeletal and gastrointestinal etiology, and was subsequently referred to cardiology clinic for evaluation of cardiac etiology for her consistent chest pain symptoms despite being on medical therapy.

**Figure 1:**
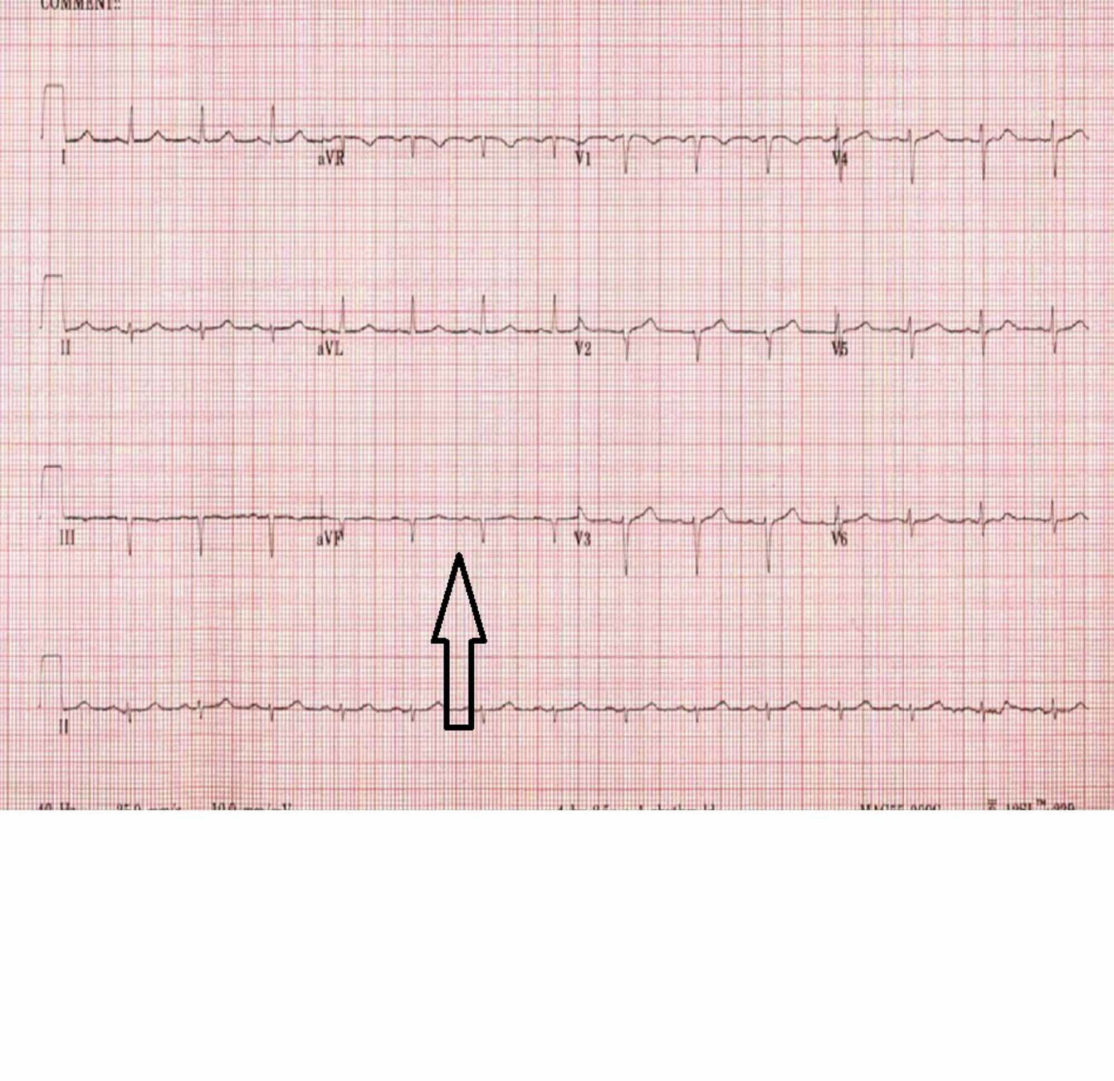
Electrocardiogram (EKG) EKG showing normal sinus rhythm, and the black arrow showing left axis deviation.

The patient reported progressive worsening of chest pain for a few months. She described radiation of the chest pain to jaw and left upper chest along with left upper extremity and shoulder area. She denied any specific association of pain with scale of activity and pain occasionally happened at rest as well. She was recently given nitroglycerin sublingual tablets for relief of her chest pain syndrome. Physical exam revealed normal cardiac findings with regular rate and rhythm. She had no murmurs and jugular venous pressure was not elevated. Electrocardiogram (EKG) showed normal sinus rhythm and left axis deviation (Figure 1).

**Figure 2:**
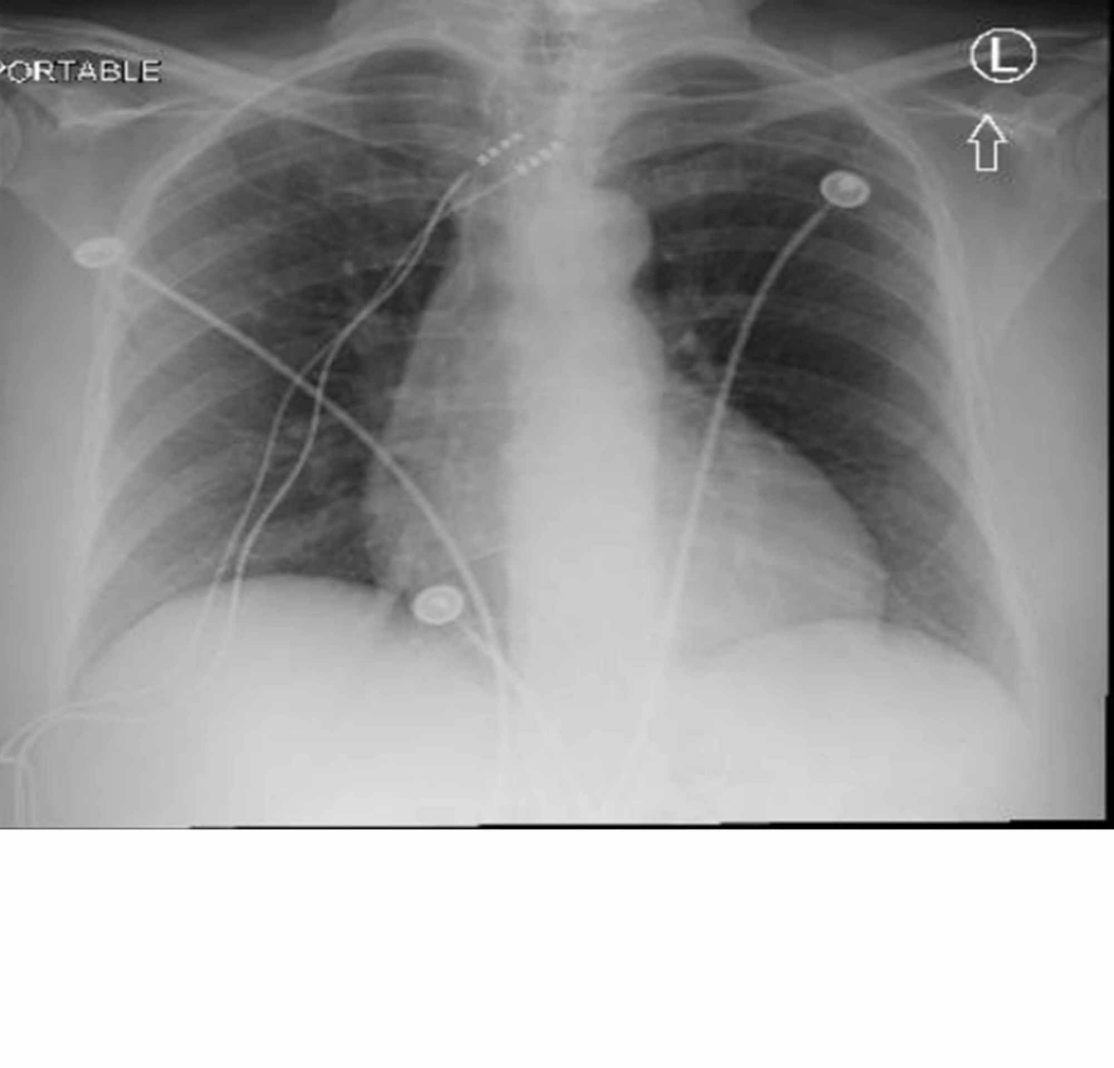
Chest X-ray Chest X-ray showing normal cardiopulmonary features.

A chest X-ray was unremarkable for any acute cardiopulmonary process (Figure 2). 

**Figure 3:**
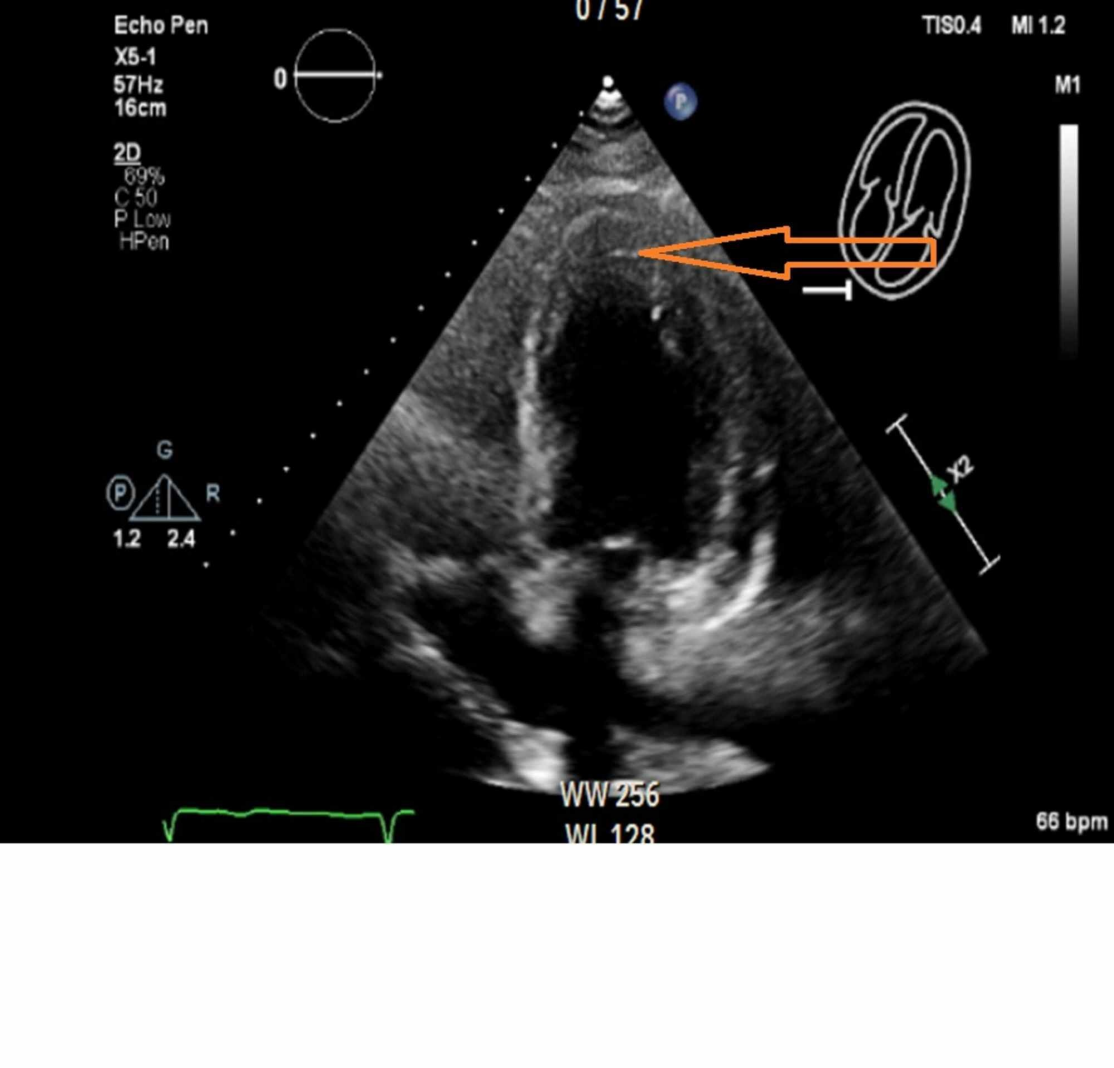
Echocardiogram The orange arrow showing akinesis of apical myocardial segment.

An echocardiogram was ordered, which showed normal left ventricular size with an estimated ejection fraction of 55%. She had concentric remodeling of the left ventricle. Echocardiogram was positive for akinesis of myocardial segments (Figure 3).

Given her abnormal echocardiogram and high pre-test probability of ASCVD and symptoms concerning for unstable angina, she was offered a left heart catheterization and coronary angiography to further delineate her coronary anatomy.

**Figure 4:**
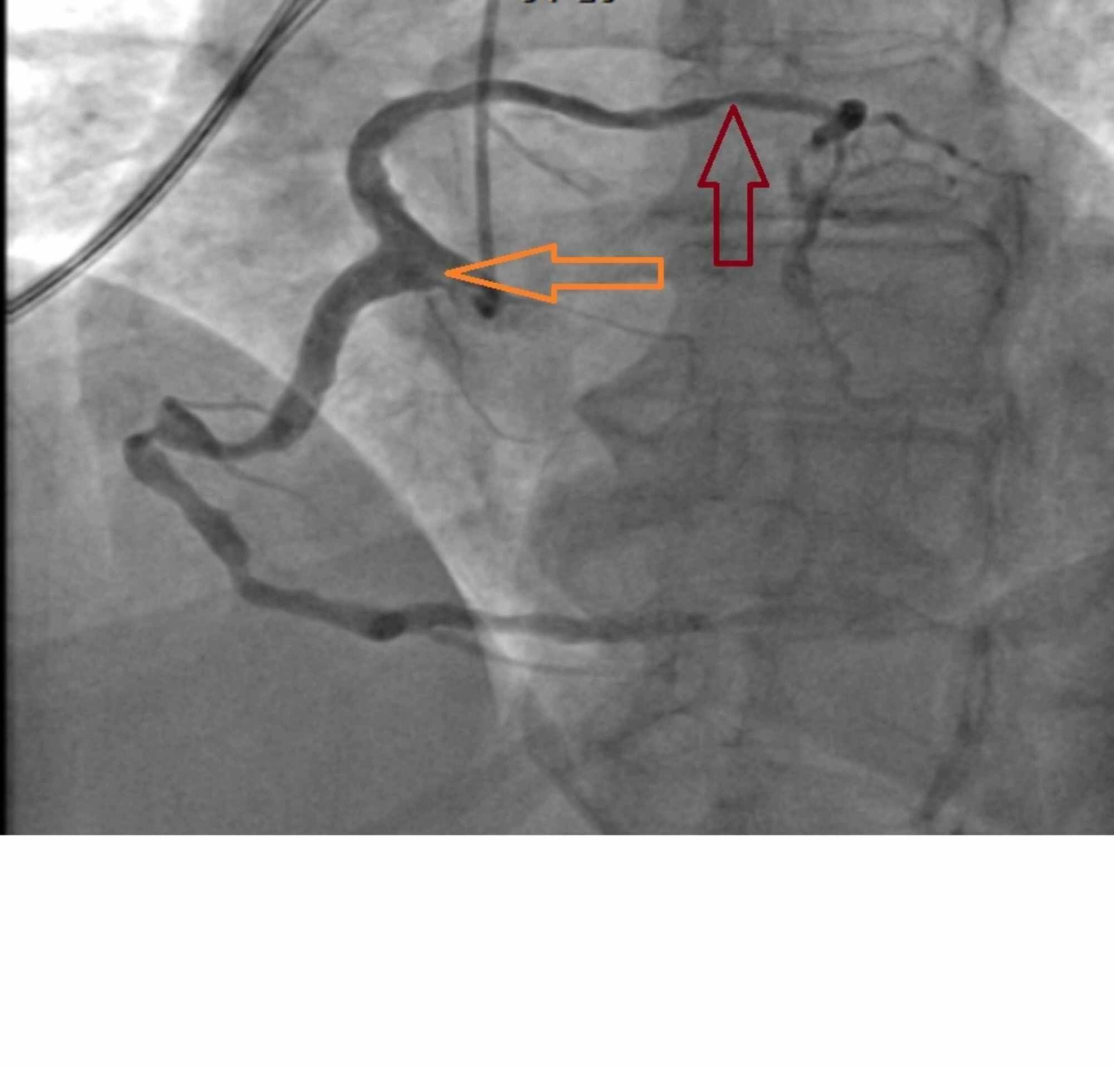
Anomalous left main coronary artery The orange arrow shows anomalous left main coronary artery originating from single right coronary ostium. The red arrow shows angiographically intermediate grade lesion in left main artery.

She was brought to the catheterization laboratory in the fasting state. Coronary artery angiography and left heart catheterization were performed via right radial artery access without complications. It revealed an anomalous left main coronary artery from single right coronary ostium (Figure 4). 

**Figure 5:**
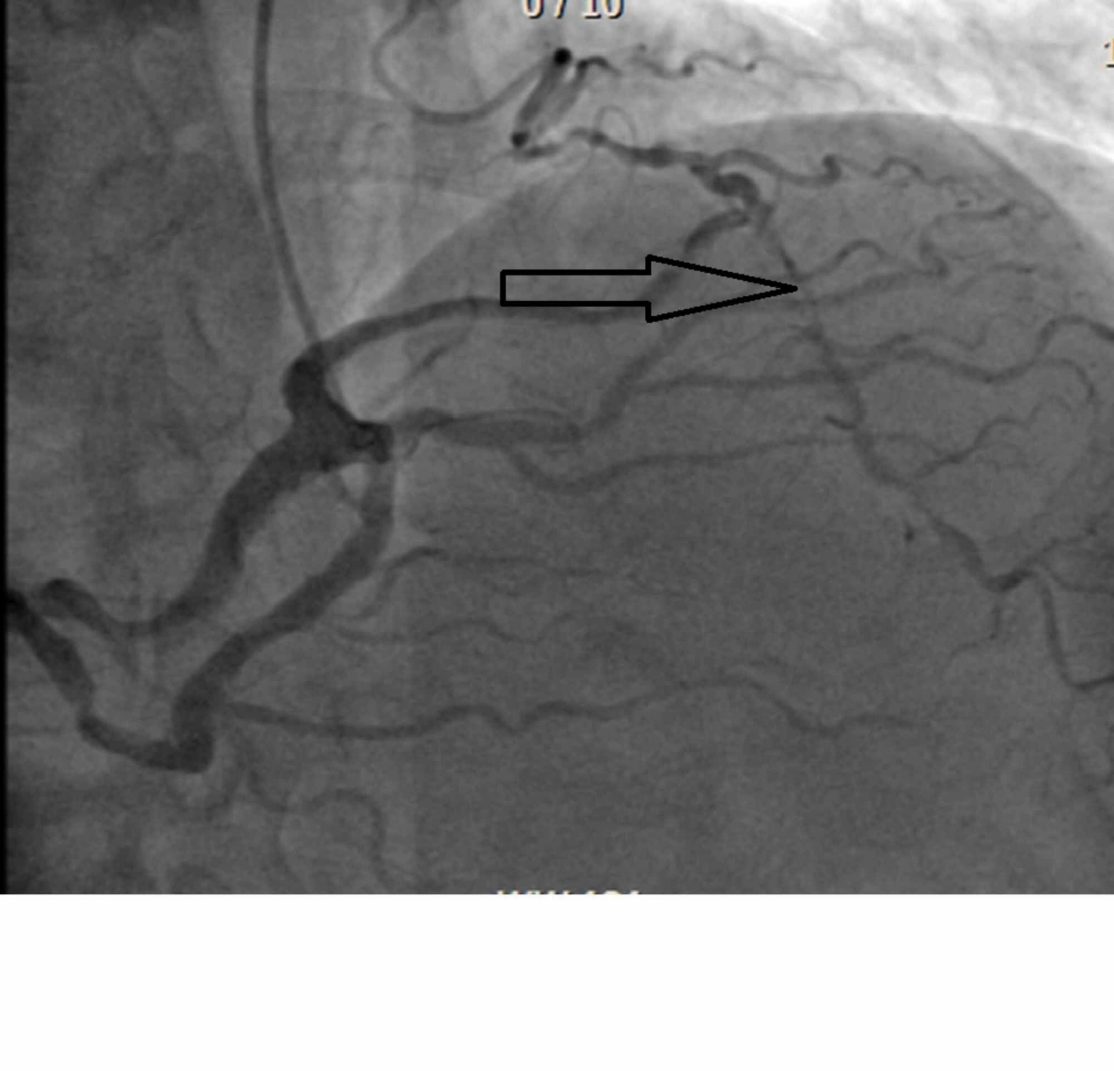
Angiogram of left interior descending artery The black arrow shows angiographically severe proximal left anterior descending artery stenosis.

It further revealed significant obstructive multi-vessel coronary artery disease involving distal left main artery, proximal left anterior descending artery, left circumflex and right coronary arteries (Figure 5).

**Figure 6:**
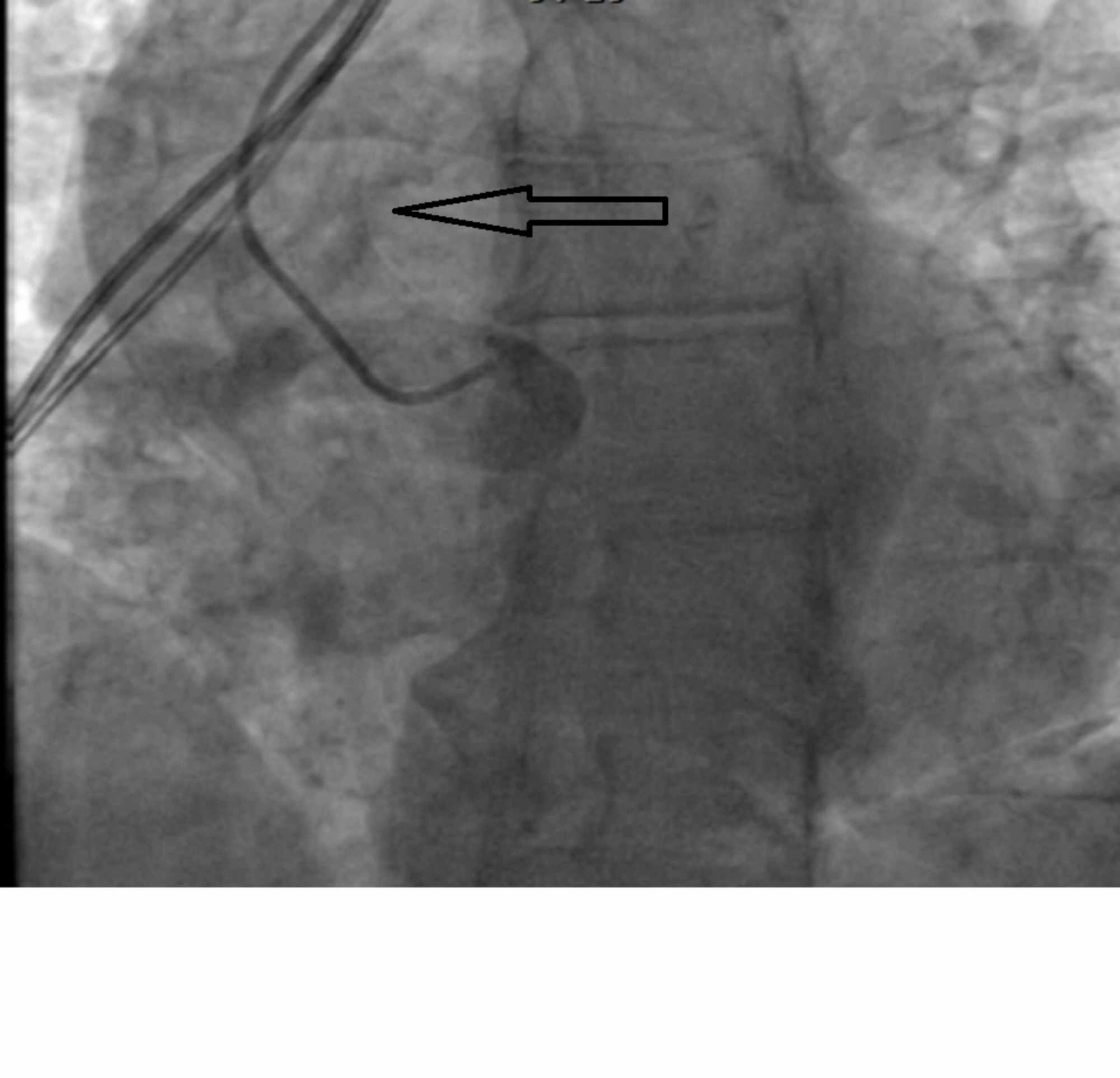
Angiogram of the aortic root The black arrow shows non-selective aortic root angiogram showing absent left coronary cusp.

The patient had a right dominant system with absent left coronary cusp (Figure 6).

She had a normal left ventricular end diastolic pressure as well. She had a high Syntax score of 34, and subsequently a Heart Team approach was pursued. She had acceptable Society of Thoracic Surgeons (STS) risk, and hence was referred for multi-vessel surgical revascularization.

## Discussion

A variety of coronary artery anomalies of varying clinical significance have been reported in contemporary literature [1,2,5,6]. And some of these anomalies can result in serious clinical outcomes including but not limited to sudden cardiac death, chest pain and arrhythmias. Obstructive atherosclerotic disease in these anomalous arteries poses a greater challenge in terms of revascularization options. Serious clinical manifestations, including acute coronary syndrome, can be particularly difficult to manage in terms of percutaneous revascularization options [7]. Although percutaneous procedures can be utilized where indicated, multi-vessel disease in anomalous coronary arteries poses a unique management challenge. 

We hereby report a unique case with a management conundrum where a patient has anomalous left main coronary artery from single right coronary ostium and obstructive multi-vessel coronary artery disease involving distal left main artery, proximal left anterior descending artery, left circumflex and right coronary arteries. While traditional percutaneous interventional catheters, coronary guide wires and equipment may be employed in such cases, stenting options can be difficult and high risk from a procedural standpoint. There should be a low threshold for Heart Team discussions, and surgical revascularization should also be considered.

Coronary lesions of intermediate severity are being increasingly investigated by physiological testing, such as fractional flow reserve (FFR) and instantaneous wave-free ratio (iFR). Various intracoronary imaging modalities, such as intravascular ultrasound (IVUS) and optical coherence tomography (OCT), have been less studied in anomalous arteries. Traditional intravascular imaging modalities, such as IVUS and OCT, may also be more difficult to be employed in anomalous arteries. Recently, Farooqi et al. reported recommendations for CT imaging and a 3D digital model for evaluation of such anomalies [8]. Similarly, other investigators have advocated for a multispecialty discussion as Heart Team approach between interventional cardiologist and cardiothoracic surgeons [9].

While moderate left main artery disease in this case could be investigated further with physiological modalities or intravascular imaging, efficacy and clinical utility of physiological testing in anomalous and long left main artery are less well established. Currently, no guidelines or expert consensus opinions exist in the literature to perform intravascular imaging via right radial access in these anatomic variations.

Our case indicates that potential utilization of intravascular imaging modalities in lesions of intermediate severity in anomalous coronary vessels including left main artery can be of significant value and may change the therapeutic and revascularization strategy subsequently. Despite the growing need of a consensus opinion, risks of potential complications during these procedures should be assessed on case-by-case basis. Jeopardizing the single coronary ostium during a percutaneous diagnostic or interventional procedure can have devastating consequences, and such procedures should be performed with extreme caution and heightened vigilance.

## Conclusions

Anomalous origin coronary arteries can present with life-threatening conditions just like in normal coronary artery disease. However, their diagnosis and management can pose a significant challenge. Evaluation of angiographically intermediate severity lesions with intracoronary imaging or physiological assessment modalities may be of potential benefit in such situations to determine best treatment approach. This is particularly of concern for patients who have a single right coronary ostium where an iatrogenic complication of percutaneous assessment or revascularization approach could be potentially fatal. 
